# Explainable Learning-Based Timeout Optimization for Accurate and Efficient Elephant Flow Prediction in SDNs

**DOI:** 10.3390/s24030963

**Published:** 2024-02-01

**Authors:** Ling Xia Liao, Changqing Zhao, Roy Xiaorong Lai, Han-Chieh Chao

**Affiliations:** 1 School of Electronic Information and Automation, Guilin University of Aerospace Technology, Guilin 541004, China; liaolx@guat.edu.cn (L.X.L.); zhaochq@guat.edu.cn (C.Z.); 2 Confederal Networks Inc., Seattle, WA 98055, USA; roy.lai@confedtech.net; 3 Department of Artificial Intelligence, Tamkang University, New Taipei City 251301, Taiwan; 4 Department of Electrical Engineering, National Dong Hwa University, Hualien 974301, Taiwan; 5 Institute of Computer Science and Innovation, UCSI University, Kuala Lumpur 56000, Malaysia

**Keywords:** explainable learning algorithms, logistic regression, Bayesian optimization, elephant flow prediction, flow entry timeout, statistics sampling

## Abstract

Accurately and efficiently predicting elephant flows (elephants) is crucial for optimizing network performance and resource utilization. Current prediction approaches for software-defined networks (SDNs) typically rely on complete traffic and statistics moving from switches to controllers. This leads to an extra control channel bandwidth occupation and network delay. To address this issue, this paper proposes a prediction strategy based on incomplete traffic that is sampled by the timeouts for the installation or reactivation of flow entries. The strategy involves assigning a very short hard timeout ($T_{initial}$) to flow entries and then increasing it at a rate of *r* until flows are identified as elephants or out of their lifespans. Predicted elephants are switched to an idle timeout of 5 s. Logistic regression is used to model elephants based on a complete dataset. Bayesian optimization is then used to tune the trained model $T_{initial}$ and *r* over the incomplete dataset. The process of feature selection, model learning, and optimization is explained. An extensive evaluation shows that the proposed approach can achieve over 90% generalization accuracy over 7 different datasets, including campus, backbone, and the Internet of Things (IoT). Elephants can be correctly predicted for about half of their lifetime. The proposed approach can significantly reduce the controller–switch interaction in campus and IoT networks, although packet completion approaches may need to be applied in networks with a short mean packet inter-arrival time.

## 1. Introduction

Packets are the fundamental unit of communication that move data on the Internet. A flow is a sequence of packets that share the same source and destination IP addresses, source and destination port numbers, and IP protocol. While flows vary in volume and type, a rather small number of flows consume the majority of the network bandwidth. These flows are elephant flows (elephants), and they typically last for a long time and consist of many packets and bytes. The remaining flows are mice flows (mice). In general, elephants are bandwidth-consuming. When two elephants share the same link, this may cause a link congestion that degrades the network’s quality of service (QoS) [1]. As the Internet becomes extremely complex due to the vast interconnection of hosts and items with widely varying intelligence and flexible QoS requirements, it is significant to accurately, efficiently, and effectively predict elephants to optimize network resources and QoS management in a global and dynamic manner.

Elephant prediction approaches in traditional networks can be applied to hosts or switches. They typically rely on the packet traffic and statistics collected by the hosts or switches to catch elephants. Since hosts and switches can only collect their local traffic in the network, there is additional cost and difficulty in collecting global traffic to optimize network resources and QoS management across the network [2].

Software-defined networks (SDNs) address this issue by predicting elephants at controllers based on global statistics [3]. SDNs have decoupled control and data planes. Controllers in the control plane can maintain global network statistics by polling the statistics from switches in the data plane or by allowing switches to report their statistics to controllers.

However, regardless of the approach controllers use to collect statistics, the statistics generated at the data plane need to be periodically transported to the control plane, resulting in additional control channel bandwidth usage [3]. Picking the right statistics update period to accurately and efficiently predict elephants at controllers is also a major challenge. The control channel is the infrastructure that connects switches to the controllers, and the data channel is the infrastructure that connects the switches to each other. Higher control channel bandwidth consumption leads to longer network latency in SDNs.

To reduce control channel bandwidth usage, current research often employs two methods: (1) deploying sampling agents on switches to sample packet traffic forwarded to controllers and (2) allowing switches to have low intelligence to sample or aggregate the statistics forwarded to controllers. However, the first method often requires software updates to the switches, and the second method lacks standardized hardware and interfaces to support programmable data planes, raising major concerns about system compatibility, interoperability, and usability in the field [4].

**Figure 1:**
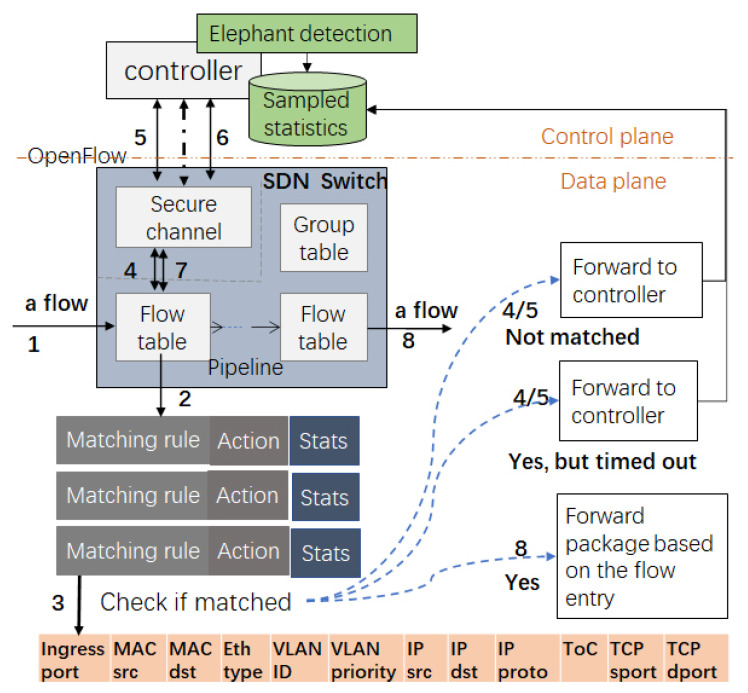
The interaction between SDN switches and controllers.

To fill this gap, this paper proposes including the timeouts of flow entries to sample packet traffic at switches, which allows the controllers to generate statistics and predict elephants. It completely avoids consuming the control channel bandwidth by moving the packet traffic dedicated to elephant prediction to the controllers. As shown in Figure 1, the flow entries in SDNs are forwarding rules generated by the controllers and installed at switches to guide the forwarding of flow packets. Flow entries on switches have timeouts to determine their lifetime. An SDN switches the forward packets to controllers if no matching flow entries are found for the packets. Accordingly, a flow forwards its first packet (in reactive flow set-up mode) and some specific subsequent packets to controllers because the matching entry has not been established or has timed out. Using flow entry timeouts to sample the packets of flows ensures that each flow forwards at least one packet to controllers, and elephants, which have more packets and longer life spans, tend to forward more packets and bytes to controllers than mice. It is possible to predict elephants based on the packets forwarded to controllers for the installation or activation of flow entries.

Such a strategy completely avoids the compatibility and deployment issues caused by inserting sampling agents into switches, as described in method (1), and the interoperability and usability issues caused by nonstandard programmable data planes, as described in method (2). Because the controllers predict elephants based on the packets received rather than on statistics that move periodically from the switches, the prediction can be made at any time a packet is received by the controllers, and thus the statistics update period at the controllers is no longer an issue. However, predicting elephants based on sampled traffic leads to a major problem in building robust elephant models based on the dataset with packet loss, specifically how dynamically adjusting the timeouts of flow entries affects the total number of packets that each flow forwards to controllers and which packets of a flow are actually forwarded. They also determine the accuracy, efficiency, and effectiveness of the elephant prediction.

This paper addresses this issue by applying explainable logistic regression (LR) to model elephants and explainable Bayesian optimization (BO) to further tune the model and timeouts. We interpret the feature selection, the learned model, and the tuned parameters. To the best of our knowledge, this is the first effort that uses the packets sampled by the timeouts of flow entries to predict elephants and apply explainable machine learning (ML) techniques for high prediction accuracy, efficiency, and effectiveness. In particular, this paper makes the following three contributions:

- Explainable LR is used to construct two elephant models, with one for all flows and the other with submodels dedicated to TCP and UDP flows.
- To maximize the prediction accuracy and efficiency and to minimize the controller–switch interactions, an optimization problem is formulated to find the best initial timeout value, the rate of increase of the timeout, and the probability used to classify an elephant. BO algorithms are used to solve the problem. Optimized parameters are interpreted.
- An extensive evaluation based on seven real traffic traces from six different networks is provided to demonstrate a generalization prediction accuracy of over 90% in about half of the lifetime. A simple packet completion approach is also provided to significantly improve the prediction accuracy while limiting the increase in network latency.

The rest of this paper is organized as follows. Section 2 and Section 3 discuss the related work and the feasibility of using packets forwarded to controllers to predict elephants, respectively. The elephant model and the timeout optimization algorithms are proposed and explained in Section 4, followed by the evaluation in Section 5 and the conclusions in Section 6.

## 2. Background and Related Work

### 2.1. SDN Architecture and Flow Entries

Unlike traditional networks, which have devices that tightly couple network control and data forwarding, SDNs have separate control and data planes, as shown in Figure 1. The SDN control plane consists of one or more controllers, and the SDN data plane includes multiple hosts and dumb switches. Dumb switches have no control capabilities and rely on SDN controllers to generate rules to guide data forwarding. Flow entries in SDNs are the forwarding rules generated by the controllers but installed in the ternary content-addressable memories (TCAMs) of the switches. Because TCAMs are power-hungry, expensive, and have limited space on the switches, each flow entry has a lifetime controlled by its timeout to effectively utilize the capacity of the TCAMs.

As illustrated in Figure 1, switches contact controllers when a flow entry needs to be created or updated. When flow entries are generated reactively, switches do not have a matching flow entry for a new flow. Therefore, a switch forwards the first packet of a flow to controllers for installation of a matching flow entry so that the subsequent packets of the flow arriving at the switch can be forwarded directly without involving the controllers again. However, flow entries have timeouts. Such timeouts can force some of the flow’s subsequent packets to be sent to controllers to reactivate the matching flow entries that have timed out. Since elephants often consist of many packets and last for a long time, they tend to forward more packets to the controllers than mice (see Section 3 for details). Such an observation motivated us to collect the packets forwarded to controllers to install or reactivate entries in order to predict elephants.

### 2.2. Flow Entry Timeouts

Flow entries generally have hard and idle timeouts, where an entry times out when the time it has been in existence and idle is greater than the set value, respectively. The SDN architecture allows controllers to configure the timeouts of flow entries. Current proposed controller solutions often give a fixed idle timeout value to the order of seconds across flows. However, the lifetime and packet inter-arrival time of flows in a network vary widely, making fixed timeouts neither resource-efficient nor performance-friendly [5].

Dynamic timeouts can address or mitigate this problem. In current research, dynamic timeouts are typically used to manage TCAMs while achieving various optimization goals [6]. Since dynamic idle timeouts were set to flows based on the longest inter-arrival time of the matched flow in history to improve flow table utilization [7], dynamic hard timeouts were set to improve the hit ratio of the flow table while reducing the number of capacity misses and occupancy [8,9]. Although Li et al. [10] used a hybrid of hard and idle timeouts to improve the same objective, Panda et al. [11] used the hybrid timeouts to enhance the durability of TCAMs during flow table overload DDoS attacks. Isyaku et al. [12] dynamically configured hybrid timeouts according to the traffic pattern based on the packet arrival time to reduce additional flow set-up requests. They adaptively allocated hybrid idle-hard timeouts to control the frequent flow set-up requests, considering the traffic pattern and flow table utilization ratio, and appropriately returned the timeout to different flows. However, to the best of our knowledge, our work is the first effort for applying ML techniques to optimize dynamic hybrid timeouts and elephant models for high elephant prediction accuracy, efficiency, and effectiveness.

### 2.3. Elephant Prediction

Elephants are predicted using a model based on the features in flow or packet levels. Flow-level models compute the size, packet counts, and duration time of flows after a sufficient number of packets in a received flow, while packet-level models compute them in the early stage of flows. Flow-level models can typically achieve a high prediction accuracy [13,14], while packet-level models may find a particular type of elephant in their extremely early stages to enable better resource management [15,16].

Elephants over SDNs can be predicted at the host [17,18], switch [17,19], and controller [20,21] of the packet statistics and traffic, which may be complete or incomplete. In general, predictions based on the former can achieve high accuracy, while predictions based on the latter may not. Because hosts originate flows and flow entries at an SDN switch update the flow counters when they are invoked, hosts and switches can maintain complete but local statistics to enable host-based and switch-based approaches, respectively. SDNs also allow controllers to maintain global and complete packet statistics by actively polling the statistics from all the switches in the networks or by allowing all the switches in the network to automatically report their statistics to the controllers to support controller-based approaches [3].

Although many prediction approaches over SDNs, such as Mahout [17], DeveFlow [22], and Hedara [20], gather complete statistics or traffic for high prediction accuracy, they have to move statistics or traffic from data to the control planes, leading to huge control channel bandwidth consumption. To avoid this, packet sampling is often used to generate incomplete statistics or traffic. For instance, Mori et al. [23] applied sFlow and determined the sampling period threshold based on Bayes’ theorem (BT) but could not reliably predict elephants until more than 10,000 packets or about 15 million bytes of a flow were received. Afaq et al. [24] enabled elephant prediction in real time by using sFlow-RT for packet sampling but could not achieve a high enough prediction accuracy. Afeq et al. [25] and Xiao et al. [26] introduced two-stage elephant prediction approaches that coordinate switches and controllers to predict elephants accurately and efficiently. Suspect elephants are determined at the switches based on the local and incomplete statistics or traffic, but complete statistics or traffic for the suspect are collected by the controllers to enable elephant prediction with higher accuracy and lower bandwidth consumption. We list these approaches in Table 1 for comparison, and to the best of our knowledge, no elephant prediction method based on the packets forwarded to controllers for flow entry installation and reactivation has been investigated.

### 2.4. Explainable Machine Learning Techniques

Since SDNs have built-in traffic statistics collection mechanisms that enable controllers to establish the global knowledge of traffic, various ML techniques have been applied to controllers based on the global knowledge to optimize flow routing [27], resource management [12,28,29], and traffic classification or prediction [2,30].

Based on the complete packet traffic, Glick et al. [31] applied a neural network (NN) to classify elephants and mice at the network edge for better flow scheduling in electric/optical hybrid data centers, and Xiao et al. [26] employed decision trees (DT) to quickly and efficiently detect elephants from the suspicious elephants identified by switches based on the statistical thresholds and flow-level features. Although Rossi et al. [32] proposed an approach similar to this work by using incomplete packet traffic to predict elephants, they applied support vector machines (SVM) to finely classify UDP flows based on aggregated packet traffic, whereas our proposed approach involves LR to model elephants and further applies BO to optimize the model and sampling parameters based on incomplete packet traffic sampled by the flow entry timeouts.

In terms of flow entry management over SDNs, Yang et al. [29] and Kannan et al. [33] applied random forest (RF) and Markov-based learning, respectively, to predict the duration of flow entries in flow tables; Al-fuqaha et al. [28] applied a deep neural network (DNN) to determine the preserved flow between elephants and mice for flow table management, and Yang et al. [34] applied multiple ML techniques to generate the best prediction model to classify flows as active and inactive to determine the correct flow to be removed. Both Li et al. [10] and this work apply the ML technique to determine the value of flow entries. However, Li et al. [10] used Q-learning to optimize the flow table resource management online, while this work applies BO to optimize the elephant model and the timeouts to maximize the elephant prediction accuracy and efficiency while maintaining the controller–switch interaction.

Many proposed ML approaches are typically black boxes with a huge parameter space and complex structure, resulting in a model that is difficult to understand, justify, and trust. The explainable artificial intelligence (XAI) technique is a way to interpret the entire model learning process. Rahman et al. [35] proposed a framework that includes explainable deep learning and edge computing to support COVID-19 diagnosis. Although Ahn et al. [36] applied a genetic algorithm (GA) to explain the feature selection for learning-based traffic classification, both Sarica et al. [37] and Mahbooba et al. [38] applied XAI to classify traffic for intrusion detection. However, the former applied the explainable RF classifier, while the latter used the explainable DT. We list these related approaches in Table 2. Our proposed approach applies statistical analysis, explainable LR, and explainable BO to select features, learn models, and tune parameters.

## 3. Feasibility Analysis

**Figure 2:**
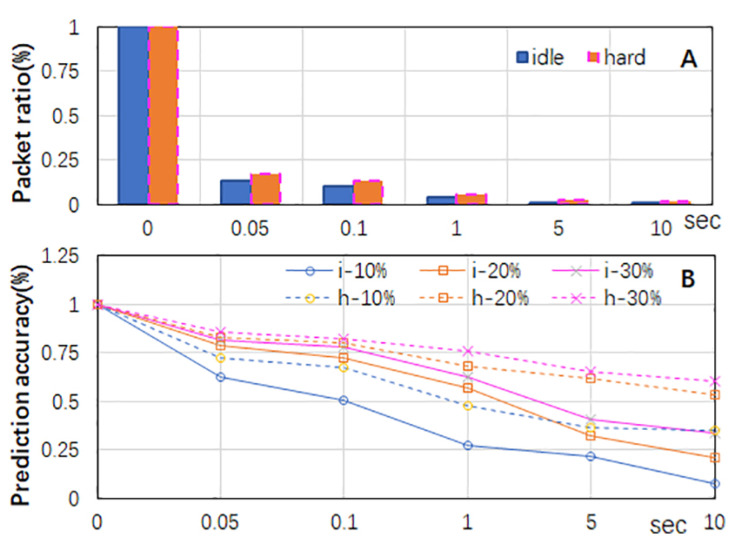
The ratio of packets sent to controllers and the elephant prediction accuracy using these packets under various timeouts. Labels with i and h indicate idle and hard timeouts were applied, respectively. (**A**) The ratio of packets sent to controllers. (**B**) The elephant prediction accuracy using the packets sent to controllers under various flow entry timeouts.

To analyze the feasibility of classifying elephants using incomplete statistics sampled by the timeouts of flow entries, we captured two packet traces from the border router connecting the campus network of Guilin University of Aerospace Technology in China to the Internet in January 2020 (TR1) and January 2021 (TR2). Each trace lasted 5 min and consisted of over 570,000 flows. We used TR1 for the feasibility analysis. We allowed the flow entries to have idle or hard timeouts and increased the values of the timeouts from 0.05 to 0.1, 1, 5, and 10 s. We computed the packet ratio using the total number of packets sent to the controllers over the total number of flow packets. As shown in Figure 2A, the packet ratio was highly dependent on the timeout of the flow entries. The shorter the timeout of the entries, the more often the entries timed out, and the more flow packets were forwarded to the controllers for entry renewal. Given the same timeout value, flows under hard timeouts sent more packets to the controllers than under idle timeouts, because idle timeouts may cause switches to forward only the first packets of elephants to the controllers, whereas hard timeouts force switches to forward the packets of flows to controllers periodically if the elephants have packets arriving at the switches frequently.

Although only less than 20% of the total number of packets of flows were sent to the controllers, we estimated the accuracy of using such packets to predict the elephants. We considered the set of flows in TR1 (*F*) and the set of flows being sent to the controllers ($F_{sent}$). We first sorted both sets in descending order by the packet count of a flow. We selected the top 10–30% of the flows from both sets and computed their intersection ($F\cap F_{sent}$) under various timeouts. Since elephants consist of more packets and last longer, it is reasonable for elephants to forward more packets to controllers than mice. Therefore, the top flows in set *F* implied the real elephants in TR1, the top flows in set $F_{sent}$ implied the predicted elephants, and $|F\cap F_{sent}\left|/|F\right|$ implied the accuracy (recall) of the elephant prediction. As shown in Figure 2B, having shorter timeouts made more top flows in both flow sets match, suggesting a higher prediction accuracy. For the same timeout value, hard timeouts led to higher prediction accuracy than idle timeouts. Accordingly, it is feasible to give flow entries short hard timeouts to achieve high elephant prediction accuracy.

However, simply configuring a short hard timeout for all the flow entries may generate a huge amount of controller–switch interaction, consuming the valuable control channel bandwidth while adding additional delay to the flow packet forwarding. To address this issue, this paper proposes applying dynamic hard-idle timeouts and optimizing their initial values and change rates using BO algorithms.

## 4. Elephant Prediction Based on Incomplete Traffic Sampled by Timeouts

This section proposes using the packets of flows forwarded to the controllers to generate or update the flow entries for prediction elephants. This completely avoids moving statistics from switches to controllers dedicated to elephant prediction. Since the flows used to predict elephants are not complete, simply applying the thresholds or models trained on complete datasets is not suitable. Since entry timeouts affect the number of packets forwarded to controllers, which further affects the elephant model used for prediction, we propose a two-step approach to maximizing elephant prediction accuracy (F1) and efficiency (*E*) while minimizing controller–switch interaction (*I*): (1) using LR based on the complete traffic dataset to learn an elephant model that inputs the selected features of a flow in TR1 and outputs the probability of that flow being an elephant, as well as (2) applying BO to find the best initial timeouts ($T_{initial}$), the rate (*r*) at which timeouts increase, and the probability threshold ($P_{elephant}$) for a flow to be an elephant.

In the rest of this section, we first analyze the features of elephants and mice and the TCP and UDP elephants in TR1 to determine the features used to model the elephants. Second, we apply LR to actually model the elephants. Third, we formulate an optimization problem that finds the best $\left[T_{initial},r,P_{elephant}\right]$ to maximize F1 and *E* while minimizing *I*. Finally, we apply BO to solve it.

### 4.1. Elephant Modeling Features

**Figure 3:**
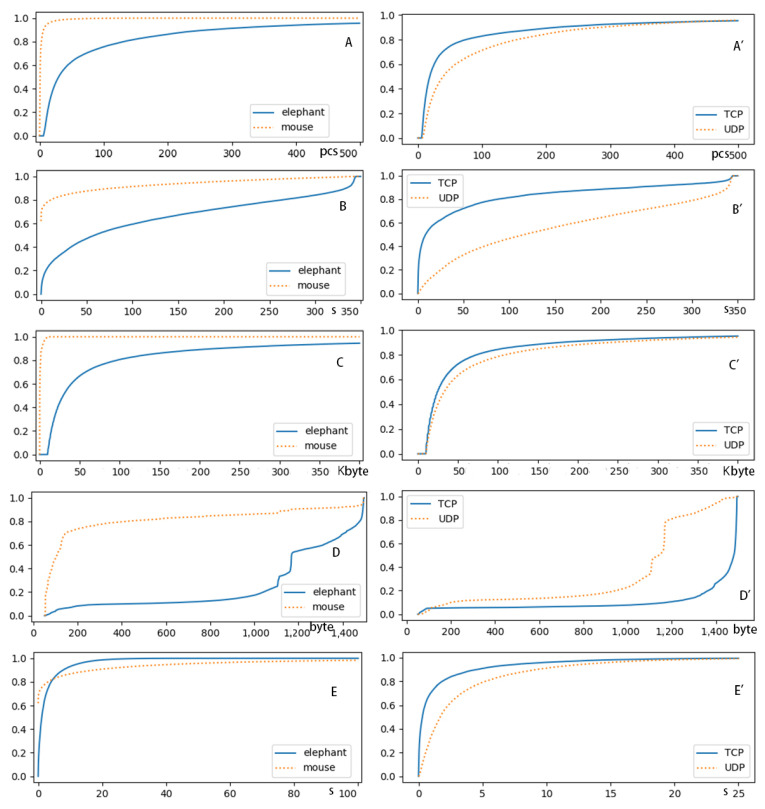
The features of flows, where the labels of elephant and mouse are the elephant flows and the mouse flows, respectively, and the labels of TCP and UDP mean the TCP and UDP elephant flows, respectively. (**A**) The CDF of packet count of elephant and mouse flows, (**B**) the CDF of flow duration of elephant and mouse flows, (**C**) the CDF of flow size of elephant and mouse flows, (**D**) the CDF of mean packet size of elephant and mouse flows, (**E**) the CDF of the mean packet inter-arrival time of elephant and mouse flows, (**A’**) the CDF of packet count of TCP and UDP elephant flows, (**B’**) the CDF of flow duration of TCP and UDP elephant flows, (**C’**) the CDF of flow size of TCP and UDP elephant flows, (**D’**) the CDF of mean packet size of TCP and UDP elephant flows, and (**E’**) the CDF of mean packet inter-arrival time of TCP and UDP elephant flows.

To determine the features of the flows used to model elephants, the flows of TR1 with more than 10,000 bytes were marked as real elephants because more than 90% of the total bandwidth usage is occupied by such flows. The cumulative distribution functions (CDFs) of the packet counts, flow size, flow duration, and mean packet size and packet inter-arrival time of all the elephants in TR1 were computed as shown in Figure 3.

We found that over 95% of the elephants had 8+ packets, while over 92.5% of the mice had 5 or fewer packets, including 62% of them having only 1 packet. While over 70% of the mice lasted less than 0.38 s, over 95% of the elephants lasted longer than 0.38 s. Although most of the elephants had flow sizes between 10,000 and 500,000 bytes, 50% and 97% of the mice had flow sizes of less than 144 and 6,000 bytes, respectively. While 80% of the mice had a mean packet size of less than 400 bytes, over 90% of the elephants had a mean packet size greater than 400 bytes, and over 80% of the elephants had a mean packet size greater than 1,000 bytes. While over 80% of the elephants had a mean packet inter-arrival time greater than 0.2 s, over 70% of the mice had one of less than 0.2 s, and 62% of the mice had one of 0 because only 1 packet was included. These demonstrate that the elephants and mice had distinct distributions in the five features, and all these features should be used to model elephants.

Since elephants can be TCP or UDP flows generated by various applications, we further analyzed the features of the TCP and UDP elephants in TR1. We found that in TR1, 63% and 37% of the elephants were TCP and UDP flows, respectively. Although TCP and UDP elephants do not have many differences in the CDF of flow size, as shown in Figure 3C’, the TCP elephants often had more packet counts, lower flow durations, and shorter packet inter-arrival times than the UDP elephants. As shown in Figure 3A’B’E’, over 50% of the TCP elephants had 19-packets, while over 70% of the UDP elephants had 19+ packets. Although over 70% of the TCP elephants lasted 50-s, over 70% of the UDP elephants lasted 50+ seconds. While over 70% of the TCP elephants had a mean packet inter-arrival time of less than 0.92 s, over 70% of the UDP elephants had one longer than that. The TCP and UDP elephants also varied in their mean packet sizes. While 80% of the UDP elephants had 800–1200 bytes per packet, over 80% of the TCP elephants had 1200–1500 bytes per packet.

Therefore, TR1 had TCP and UDP elephants that differed greatly in features other than flow size. Given two networks, they may have similar feature distributions for dedicated TCP or UDP elephants, since TCP and UDP elephants on two networks are often generated by similar types of applications. However, the overall elephant distribution between two networks can be widely different due to the various ratios of TCP and UDP elephants that make up the network. Accordingly, an elephant model trained by TR1 may not be able to achieve high generalization accuracy over another network. These observations motivated us to train dedicated models for TCP and UDP elephants to improve the robustness of the models.

Since we used complete packet traffic to train the elephant models but predicted elephants based on the packet traffic sampled by flow entry timeouts, the two packet traffic datasets had a large difference in packet count and mean packet inter-arrival time. Therefore, we chose the accumulated flow duration (fduration), flow size (fsize), and mean packet size (psize) to model the TCP and UDP elephants and improve the robustness of the models.

### 4.2. Explainable Logistic Regression for Elephant Modeling

LA is a classification algorithm used to assign observations to a discrete set of classes. We chose it for its simplicity and explainability. In our case, we had two classes of flows: elephants and mice. We labeled elephants as one and mice as zero. LA generates a hyperplane, as shown in Equation (1), to separate the samples in the dataset and further transforms the output to a probability using the logistic sigmoid function, as shown in Equation (2). This probability is then further mapped to elephants or mice using a threshold $P_{elephant}$, where *F* is the set of flows in TR1 and *i* is a flow in the set *F*:

(1) $$Z_{i}=W_{i}X_{i}^{T}+b$$

(2) $$P\left(Z_{i}\right)=\frac{1}{1+e^{-Z_{i}}}$$

(3) $$X_{i}=\left[psize_{i},fsize_{i},fduration_{i}\right]$$

(4) $$W_{i}=\left[w1_{i},w2_{i},w3_{i}\right]$$

Let $P_{elephant}$ be 0.5. Then, the flow *i* is classified as an elephant if $P(Z_{i})$ is greater than $P_{elephant}$. We applied the built-in LA function in Python to learn $W_{i}$ for the feature $X_{i}$ and the parameter *b*. As illustrated in Equation (3), for each flow i∈F, the features of the mean packet size $psize_{i}$, flow size $fsize_{i}$, and flow duration $fduration_{i}$ are the inputs of LA, and $w1_{i}$, $w2_{i}$, and $w3_{i}$ are the weights of the features. We consider two scenarios: (1) training a model for all elephants and (2) training a model with submodels dedicated to TCP and UDP elephants.

As shown in Table 3, we have $W_{i}=\left[0.000166101,0.00322325,-0.00011509\right]$ and b=−32.2119 for scenario 1. In scenario 2, we have $W_{i}=\left[-0.00029942,0.00283122,-0.00342648\right]$ and b=−27.2731 for the TCP elephants and $W_{i}=\left[-0.00043848,0.00260208,-0.003015\right]$ and b=−25.6452 for the UDP elephants. It can be seen that the flow size feature had the highest weight in both scenarios, implying that the total bytes of a flow are key for elephant classification. Both models achieved a prediction F1 score of 99+% over TR1.

To roughly estimate the accuracy of such models over the sampled dataset, we used a fixed hard timeout of 0.00001 s to sample TR1. We set $P_{elephant}=0.5$. The prediction F1 scores of both models were around 0.89. Although the prediction accuracy can be measured by the recall, precision, and F1 score, we chose the F1 score to balance the recall and precision simultaneously. It is obvious that the two models had lower prediction F1 scores over the sampled dataset because sampling leads to packet loss, which decreases the accuracy of the model used to predict elephants. Accordingly, we needed to optimize the timeouts of the flow entries as well as the elephant model to achieve a high prediction accuracy.

### 4.3. Optimization Problem Formulation

In our proposed approach, the prediction can be performed at any moment when the controllers receive a packet of flows forwarded by switches. Since the traffic used to predict elephants is sampled by the timeouts of the flow entries, a major challenge is to tune the elephant model and the sampling timeouts. Accordingly, the elephant model generated by the complete traffic dataset, as listed in Table 3, and the packet sampling timeouts should be optimized.

As shown in Equations (1) and (2), given the packet count, flow size, and duration of flow *i* to LR, it outputs the probability $P(Z_{i})$. By comparing $P(Z_{i})$ with the given threshold $P_{elephant}$, the model classifies the flow *i* as an elephant if $P\left(Z_{i}\right)>P_{elephant}$ and as a mouse otherwise. Since the chosen features are not significantly affected by sampling, the CDF of such features over the sampled dataset has a high probability of having a similar shape to that over the complete dataset. Therefore, we believe that simply adjusting the probability threshold $P_{elephant}$ instead of the parameters $W_{i}$ and *b* of the model trained on a complete dataset can achieve high prediction accuracy over the sampled dataset. Therefore, we formulated an optimization problem that finds the best initial timeout ($T_{initial}$), timeout increase rate (*r*), and probability threshold ($P_{elephant}$) to maximize the elephant prediction accuracy (F1) and efficiency (*E*) while minimizing the network delay (*L*).

Given $\left[T_{initial},r,P_{elephant}\right]$ for each flow i∈F, a flow entry starts with a hard timeout of $T_{initial}$. When the timeout expires, the value of the timeout increases to *r* times the current until the flow is classified as an elephant or out of its lifetime. When a flow is classified as an elephant, its flow entry is configured with an idle timeout of 5s. In particular, when a flow packet is forwarded to the controllers, Equations (1) and (2) are used to compute the probability that this flow is an elephant. Let $label_{i}$ be the label of flow *i*. It is one if flow *i* is a real elephant and zero otherwise. We let $plabel_{i}$ be the label of flow *i* predicted by the model. Since the controllers keep making predictions for each flow as the flow accumulates, $plabel_{i}$ is the final decision made by the controllers. Then, the F1 score (F1) of the target prediction can be computed using Equation (5), where Rec and Pre are the recall and precision, which can be computed using Equations (6) and (7), respectively.

The elephant prediction efficiency (*E*) represents how long an elephant has lived when it is correctly predicted. Let $Telephant_{i}$ be the cumulative time duration in which flow *i* is predicted to be an elephant and $T_{i}$ be the lifetime of flow *i*. Then, $Telephant_{i}/T_{i}$ computes the prediction efficiency of flow *i*, and the overall prediction efficiency (*E*) is the average prediction efficiency for all real elephants that are correctly predicted, as shown in Equation (8).

Given $\left[T_{initial},r,P_{elephant}\right]$, we simply use the total number of packets of flows forwarded to the controllers ($C_{\left[T_{initial},r,P_{elephant}\right]}$) over the total number of packets of flows forwarded to the controllers under an idle timeout of 1 s ($C_{baseline}$) to represent the increase in network latency (*L*), as formulated in Equation (9), since the greater the volume of packets forwarded to the controllers, the more extra network forwarding latency is added.

To compute $C_{\left[T_{initial},r,P_{elephant}\right]}$, for each flow i∈F, we have a binary parameter $b_{ij}$. Let $b_{ij}$ be one if the *j* packet of flow *i* is forwarded to the controllers and zero otherwise. If $t_{ij}$ is the time at which the *j*th packet of flow *i* arrives at the switch, $tactivate_{i}$ is the most recent activation time of the flow entry of flow *i*, and $Tcurrent_{i}$ is the current hard timeout value of the flow entry of flow *i*, then the *j*th packet of flow *i* is forwarded to the controllers if $t_{ij}-tactivate_{i}>Tcurrent_{i}$, and $C_{\left[T_{initial},r,P_{elephant}\right]}$ can be calculated using Equation (10). $K_{i}$ is the total number of packets of flow *i* forwarded to the controllers. $C_{baseline}$ can also be calculated using Equation (10) if we let $tcall_{i}$ be the last call time of the flow entry of flow *i*, and let $b_{ij}$ be one if $t_{ij}-tcall_{i}>1$ and zero otherwise:

(5) $$F1=\frac{2\times Pre\times Rec}{Pre+Rec}$$

(6) $$Rec=\frac{\sum_{i\in F}plabel_{i}\times label_{i}}{\sum_{i\in F}label_{i}}$$

(7) $$Pre=\frac{\sum_{i\in F}plabel_{i}\times label_{i}}{\sum_{i\in F}plable_{i}}$$

(8) $$E=\frac{\sum_{i\in F}plable_{i}\times label_{i}\times \frac{Telephant_{i}}{T_{i}}}{\sum_{i\in F}plable_{i}\times label_{i}}$$

(9) $$L=\frac{C_{\left[T_{initial},r,P_{elephant}\right]}}{C_{baseline}}$$

(10) $$C_{\left[T_{initial},r,P_{elephant}\right]}=\sum_{i\in F}\sum_{j=2}^{K_{i}}\left(1+b_{ij}\right)$$

Let *S* be the entire domain of $\left[T_{initial},r,P_{elephant}\right]$. The proposed optimization problem can be formulated to find the best $[T_{initial},r,P_{elephant}]\in S$ such that the elephant prediction inaccuracy (1/F1), efficiency (*E*), and network latency increase (*L*) are minimized. Since this optimization problem has three conflicting objectives, the best solution to the problem is not unique. We then give weights [0.6,0.2,0.2] to the objectives [1/F1,E,L], respectively, and the proposed optimization problem can be simplified as shown in Equation (11), where the final objective function $f(\left[T_{initial},r,P_{elephant}\right])=0.6/F1+0.2E+0.2L$, as shown in Equation (12). All the symbols used in this paper are listed in Table 4:

(11) $${\left[T_{initial},r,P_{elephant}\right]}^{*}=argMin_{[T_{initial},r,P_{elephant}]\in S}f\left(\left[T_{initial},r,P_{elephant}\right]\right)$$

(12) $$f\left(\left[T_{initial},r,P_{elephant}\right]\right)=\frac{0.6}{F1}+0.2E+0.2L$$

### 4.4. Applying Explainable Bayesian Optimization

It is time-consuming for the proposed optimization problem to exhaustively search the entire solution set (*S*) to find the best solution. Gradient descent-based optimization approaches can quickly find the global optima but are not suitable for solving the proposed problem because the proposed problem is discrete, and its derivative cannot be easily achieved. Grid search, random search, and genetic search approaches are also unsuitable because they are either too computationally expensive or cannot generate approximate solutions of sufficient quality.

BO is a search mechanism based on BT. It performs searches efficiently and effectively. Before applying BO to solve our proposed problem, we should (1) represent the parameters as a vector; (2) define the search space; (3) formulate the objective function; and (4) calculate the cost over the objective function.

The solution is represented as $\left[T_{initial},r,P_{elephant}\right]$. Since BO requires a continuous solution domain, we let $T_{initial}$ be a real number in $\left[T_{shortest},5\right]$, and $T_{shortest}$ is a short value that should consider the shortest packet inter-arrival time of the elephants in the training dataset (we let $T_{shortest}=0.00001$ s in TR1). We let *r* be a real number in [1, 5], since we preferred the timeouts to grow continuously to reduce the total number of packets of a flow forwarded to the controllers. We let $P_{elephant}$ be a real number in [0.2,0.9] to adjust the probability of elephant prediction without actually changing the elephant model. The objective function is 0.6/F1+0.2E+0.2L, where F1, *E*, and *L* are formulated as shown in Equations (5), (8) and (9), and the cost is the output of the objective function.

Let A and B be two events, where P(B|A), P(A), and P(A|B) refer to the likelihood, prior, and posterior probabilities, respectively. According to BT, we have P(A|B)=P(B|A)×P(A) (given P(B) to be normalized), which provides a framework to quantify the beliefs about an unknown objection function given samples that form the domain and their evaluation via the objective function. In our case, a sample refers to $s_{i}=\left[T_{initial},r,P_{elephant}\right]$, and it is estimated using the objective function ($f(s_{i})=0.6/F1+0.2E+0.2L$), while $f(s_{i})$ also refers to the cost of $s_{i}$. The samples and their costs were collected sequentially to form data $D=[s_{1},f\left(s_{1}\right),...,s_{n},f\left(s_{n}\right)]$ to define the prior P(f). The likelihood P(D|f) is defined as the probability of observing data *D* given P(f) and keeps changing as more observations are collected.

The posterior (P(f|D)=P(D|f)×P(f)) represents what we have known about the objective function. It is an approximation of the objective function and can be used to estimate the cost of different candidate samples that we may want to evaluate. It is a surrogate objective function that probabilistically summarizes the conditional probability of the objective function *f*, given the available data (*D*) or P(f|D). Here, we chooe Gaussian process regression (GPR) to estimate *f*, since it is widely used and is capable of efficient and effective summarization of a large number of functions and smooth transition as more observations are made available to the model. Based on the estimate, an acquisition function is involved in finding the samples in the search space that are most likely to pay off. As additional samples and their evaluation via the objective function are collected, they are added to the data *D*, and the posterior is then updated. This process is repeated until the given number of iteration is exhausting. Our proposed BO algorithm is illustrated in Algorithm 1.
**Algorithm 1** BO algorithm for our proposed problem1:INPUT: the number of iterations, the set *S*, and the data D=∅2:OUTPUT: the best $\left[T_{initial},r,P_{elephant}\right]$3:initialize *D*4:compute the GP over *D*5:pick a new $\left[T_{initial},r,P_{elephant}\right]$ using acquisition function6:compute its cost7:update *D*8:**if** the number of iterations has been exhausted **then**9:    go to 210:**else**11:    go to 412:**end if**

The algorithm was applied to both scenarios 1 and 2, as listed in Table 3. While a set of ${\left[T_{initial},r,P_{elephant}\right]}^{*}$ was optimized for all flows in scenario 1, two dedicated sets of ${\left[T_{initial},r,P_{elephant}\right]}^{*}$ were optimized for the TCP and UDP flows separately in scenario 2. The results show that [0.00001,1.047,0.7] was best for scenario 1 and [0.00001,1,0.566] was best for the TCP and UDP elephants in scenario 2. This implies that giving the fixed hard timeout of 0.00001 s over TR1 or slightly increasing the timeout value to 1.047 times the current timeout once the flow entries are timed out (until the flows are predicted to be elephants or out of their lifetime) can minimize the elephant prediction accuracy, efficiency, and the increase in network latency. After a flow was predicted to be an elephant, its flow entry was switched to an idle timeout of 5 s.

**Figure 4:**
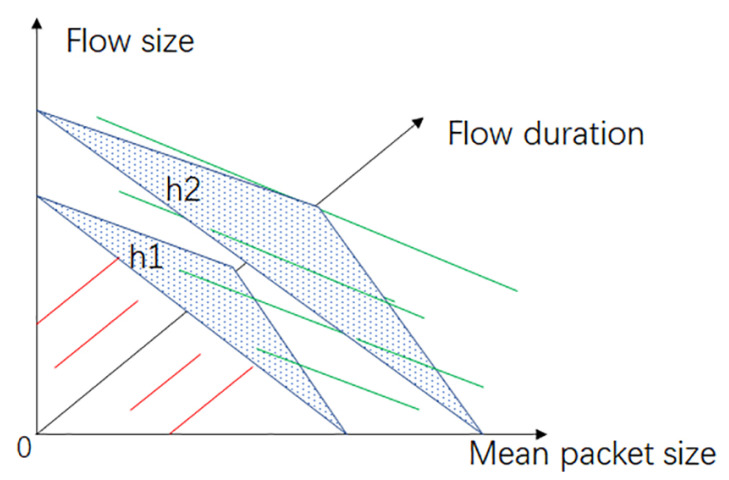
The hyper-plane used to predict elephants in logistic regression, where h1 is the hyper-plane under a probability of 0.5 and h2 is the hyper-plane under probabilities greater than 0.5.

It should be noticed that the probability threshold $P_{elephant}$ was adjusted to 0.7/0.566 from 0.5. In LR, the flows were placed with P(Z)=0.5 on plane h1, as shown in Figure 4. In the trained model, the flows with P(Z)>0.5 were predicted to be elephants, and they were placed on top of plane h1. Adjusting $P_{elephant}$ to 0.7/0.566 from 0.5 implies that the hyper-plane that separated the elephants and mice moved to h2. This is because the traffic sampled by the timeouts was incomplete, and a flow needs more time to accumulate packets so that the features generated on the incomplete traffic can have a similar value to those based on complete traffic. Moving hyper-plane h1 to h2 indicates the increase in the probability threshold, as shown in Figure 4.

## 5. Evaluations

In this section, we collect seven traces of campus (TR1, TR2, Simpleweb [39], UNIBS [40], and UNIV1 [41]), backbone (MAWI, http://www.fukuda-lab.org/mawilab/, accessed on 20 October 2023), and IoT (IoT [42]) networks from different countries, as listed in Table 5. We first evaluate the robustness of the proposed elephant model and the parameters optimized by BO algorithms. Second, we analyze how the parameters affected the three objectives. Third, we estimate the overall performance of the BO algorithms. Finally, we discuss the weakness of the proposed approach, the methods to overcome the weakness, and how this approach can be applied to other scenarios.

### 5.1. Robustness

This subsection applies the two sets of parameters optimized by BO in Section 4.4 ($\left[T_{initial},r,P_{elephant}\right]$ can be [0.00001, 1.047, 0.7] or [0.00001, 1, 0.566]) and computes the values of the three objectives over the seven datasets as listed in Table 5.

**Figure 5:**
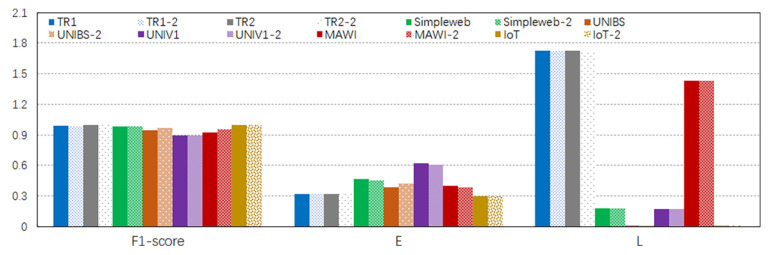
The three objectives given $\left[T_{initial},r,P_{elephant}\right]$ in two cases. The labels without −2 indicate the values for scenario (1) (a general model for both TCP and UDP elephants). The labels with −2 indicate the values for scenario (2) (dedicated models for TCP and UDP elephants).

As shown in Figure 5, although both two-parameter sets achieved high generalization accuracy over seven datasets, using dedicated TCP and UDP elephant models obtained a prediction F1 score that was about 0.01 higher than using the same model for both the TCP and UDP flows. In high-performance networks such as UNIBS, UNIV1, and MAWI, having an F1 score of 1% higher implies that hundreds or thousands more elephants can be predicted correctly, leading to better network performance and resource management. Although the elephant predictions over all datasets achieved an F1 score of over 90%, the F1 score over UNIV1 was the lowest in both scenarios. More research should be carried out in the future to further improve the robustness of the elephant model.

All the datasets except UNIV1 could predict elephants in half of the elephants’ lifetime. In practice, this implies that the proposed approach can detect elephants in their early stages so that the routes of elephants can be optimized for their subsequent packets to achieve better resource utilization and performance management. UNIV1 had lower prediction efficiency because its elephants had a shorter lifetime and a smaller mean packet size, causing late elephant prediction.

Regarding the third objective of minimizing the increase in network latency, we calculated the ratio of packet counts forwarded to the controllers. It can be seen that TR1 and TR2 forwarded more packets to the controllers than the other campus datasets. This is because (1) they had packets arriving much faster than the other campus networks, as listed in Table 5, and (2) they had more UDP elephants (ratio) than the other datasets, while UDP elephants typically have longer packet inter-arrival times and lifetimes than TCP elephants. We noticed that the ratio of packet counts forwarded to the controllers over MAWI was lower than those over TR1 and TR2, although MAWI was the trace of the carrier-level network. This may have been caused by the packets with sizes greater than 1,500 bytes in MAWI. Such an observation implies that some small packets were aggregated by the network interface cards to improve the overall performance, and the real packet arrival frequency should be much higher than that calculated by the trace. This leads to a decreased ratio of packet counts forwarded to the controllers.

### 5.2. Sensitive Analysis

BO requires $T_{initial}$, *r*, and $P_{elephant}$ to have continuous domains, and the domains of $T_{initial}$, *r*, and $P_{elephant}$ can affect the output of BO. Since the elephant prediction accuracy had the weight of 0.6 in the proposed objective function, and the probability threshold affected the elephant prediction accuracy, we varied the domain of $P_{elephant}$ and saw how it affected the output of BO. Specifically, we let the domain of $P_{elephant}$ be [0.2, 1], [0.2, 0.9], [0.2, 0.8], and [0.2, 0.7] and applied BO over all the datasets, as listed in Table 5. The parameters optimized by BO are listed in Table 6, where parameters 1, 2, 3, and 4 used the same model for both the TCP and UDP flows, while parameters 5, 6, 7, and 8 applied dedicated models for the TCP and UDP flows. The models are listed in Table 3.

It can be seen that varying the domain of $P_{elephant}$ did change the final *r* and $P_{elephant}$ output by BO, but $T_{initial}$ remained unchanged at 0.00001 s, implying that giving a shorter $T_{initial}$ to the sample traffic could achieve a higher prediction accuracy, since the packet loss generated by sampling would be reduced. As the domain of $P_{elephant}$ varied, the *r* and $P_{elephant}$ optimized by BO changed, causing a large difference in the prediction accuracy, efficiency, and the increase in network latency.

**Figure 6:**
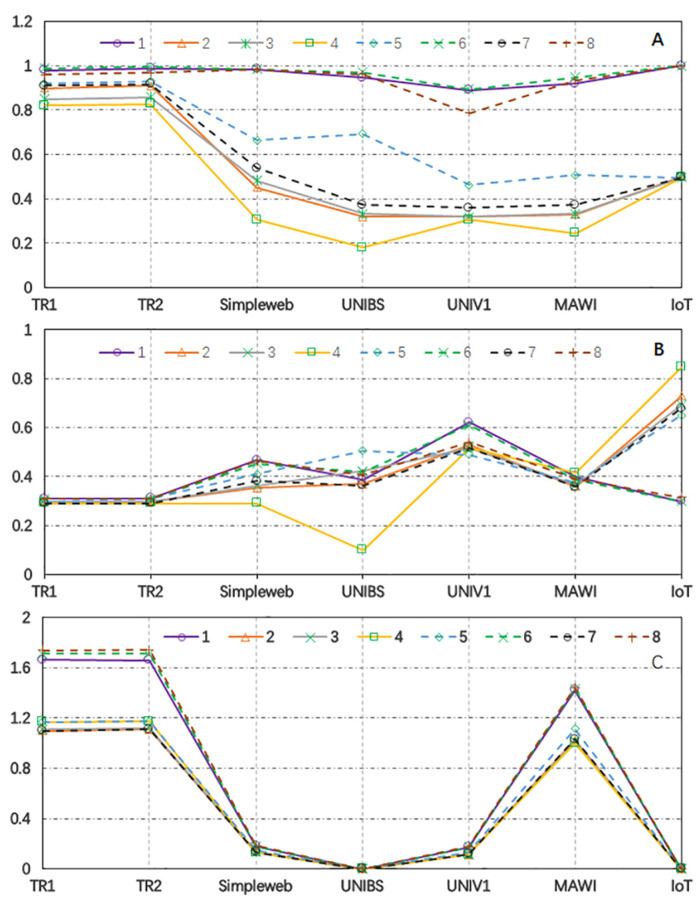
The three objectives given $\left[T_{initial},r,P_{elephant}\right]$ for parameters of 1–8 as listed in Table 6. (**A**) The F1 score of elephant prediction. (**B**) The elephant prediction efficency. (**C**) The network latency increase.

As shown in Figure 6A, parameters 1, 6, and 8 had higher generalization F1 scores than the others. Although parameters 1, 6, and 8 had $P_{elephant}$ values of 0.7, 0.9, and 0.2, respectively, their *r* values had an equal value or were slightly greater than 1, whereas the values of *r* for the other parameters were much larger. This indicates that having a short and uniform sampling period for flow packets has a significant chance for the features of flows to maintain the same distribution, which leads to higher generalization accuracy.

As shown in Figure 6B, the elephant prediction efficiencies of Simpleweb, UNIBS, and IoT were highly impacted by the parameters. However, such datasets achieved higher prediction efficiency when using parameters 1, 6, and 8 because such parameters forced more flow packets to be forwarded to the controllers in the early stages of the flows. The IoT dataset could not predict elephants in their early stages when having *r* equal to or greater than 1.8, because the elephants in the IoT dataset had quite small packet sizes and long packet inter-arrival times.

As shown in Figure 6C, although Simpleweb, UNIBS, UVIC1, and the IoT forwarded a rather small quantity of traffic to the controllers, the volume of traffic forwarded by TR1, TR2, and MAWI to the controllers was 0.9–1.8 times the base line. This is because TR1, TR2, and MAWI have shorter packet inter-arrival times, causing more flow packets to be forwarded. The same short $T_{initial}$ with a larger *r* can reduce the quantity of traffic forwarded to the controllers, but the generalization prediction accuracy over networks can be decreased. It can also be noticed that the same approach over TR1 and TR2 forwards a higher ratio of packets to the controllers than MAWI, though TR1 and TR2 were the traces of campus networks, while MAWI was the trace of a carrier-level network. This is because TR1, TR2, and MAWI are from 10G+ networks. However, we carefully configured the network interface cards for TR1 and TR2 to avoid generating packets with sizes greater than 1,500 bytes, while MAWI’s was not. Since such large packets (with packet sizes greater than 1,500) do not reflect the real behaviors of packets at links, the prediction F1 score, network efficiency, and the increase in network latency over MAWI may not represent the real performance of the models in reality.

In summary, the strategy that set a short enough $T_{initial}$ to flow entry timeouts and remain unchanged until identifying flows as elephants, followed by changing the flow entries of the elephants to an idle timeout of 5 s, could achieve high generalization accuracy across seven different datasets. The generalization accuracy is highly sensitive to the rate at which timeouts increase (*r*). Maintaining a rate of one or slightly above one could keep the prediction F1 score above 90% over various networks. The prediction efficiency and the increase in network latency were both impacted by the value of *r* and the features of the network. For instance, the prediction efficiency was highly dependent on the flows’ mean packet size, and the increase in network latency was heavily determined by the flows’ mean packet inter-arrival time. Therefore, the IoT dataset showed poor prediction efficiency for certain parameters, and TR1, TR2, and MAWI forwarded more packets to the controllers when a higher prediction F1 score was demanded.

### 5.3. Performance of BO

The BO algorithms used in this work are based on the function BayesianOptimization in the package of bayes_opt in Python. This function applies GPR with the Mertin kernel to construct a posterior distribution that best describes the optimized cost function. The function was set to the default parameters. For example, the number of steps in BO was 25, the number of initial points was 5, and they were randomly selected in the domains, while the acquisition function used was the upper confidence bound. The algorithms were executed on a laptop with an Intel i7-8750H CPU running at 2.20 GHz and 8 GB of RAM.

Let *m* (|F|=m) flows in the dataset, where each flow has *n* packets on average and the time complexity of solving Equation (11) is O(mn). Let the number of steps of BO be *a* and the number of initial points be *b*, while the time complexity of the BO algorithms is O(abmn).

Compared with a grid search, which may take several days to find the best parameters from the parameter domain, the overall running time of the BO algorithms was about 3 h, regardless of whether they used the same or dedicated models for TCP and UDP elephants over TR1. Using a computer with a stronger computation capability, applying a dataset with fewer flows, and reducing the number of iterations could decrease the time cost. However, increasing the number of iterations did not ensure better parameter outputs, although the overall time cost was increased. In fact, we did not find better parameters when increasing the number of iterations to 60.

We also let the acquisition function be the experience improvement and the probability of improvement, alongside the upper confidence bound. We applied the BO algorithm to the TR1 dataset. We did not find a significant improvement in the quality of the parameters or the overall time cost. Since we let BO randomly generate five initial points in their domains, BO may output various parameters in different runs. In this work, we let BO run several times for each set of inputs and chose the best outputs.

### 5.4. Discussion

#### 5.4.1. Bandwidth Usage

As shown in Figure 6C, our proposed approach forwarded more packets to the controllers and consumed more control channel bandwidth than the base line over TR1, TR2, and MAWI. However, it should be noticed that our proposed approach did not need to move traffic or statistics from the switches to the controllers dedicated to elephant prediction. In fact, typical controller-based elephant prediction approaches not only consume control channel bandwidth to forward packets from switches to controllers for installation and reactivation flow entries, but they also move network traffic or statistics from switches to controllers periodically to predict elephants. According to DevoFlow [17], a switch can return 1.3 million bytes of statistics to the controllers. Let the controllers poll the switch twice per second [20]. Then, 780 million bytes (1.3 × 2 × 60 × 5 = 780) of statistics are forwarded to the controllers in 5 min. Since the mean packet size of TR1 is 800 bytes, 780 million bytes of statistics is roughly 975,000 ($780\times 10^{6}/800=$ 975,000) packets, which is roughly 0.54 times the base line (the number of packets forwarded to the controllers under an idle timeout of 1 s). In fact, the real control channel bandwidth usage over TR1 for a typical controller-based approach should be 1+0.54n of the base line, where *n* is the number of switches in a network, because each switch in the network has to send its flow statistics to the controllers periodically. Since our proposed approach only requires the first switch along the route of a flow to forward packets to the controllers, while the real control channel bandwidth usage for our proposed approach is less than 1.8 times the base line (as shown in Figure 6C), the real control channel bandwidth usage for our proposed approach is lower than that of a typical controller-based approach over a network consisting of 1+ switches.

#### 5.4.2. The Increase in Network Latency

**Figure 7:**
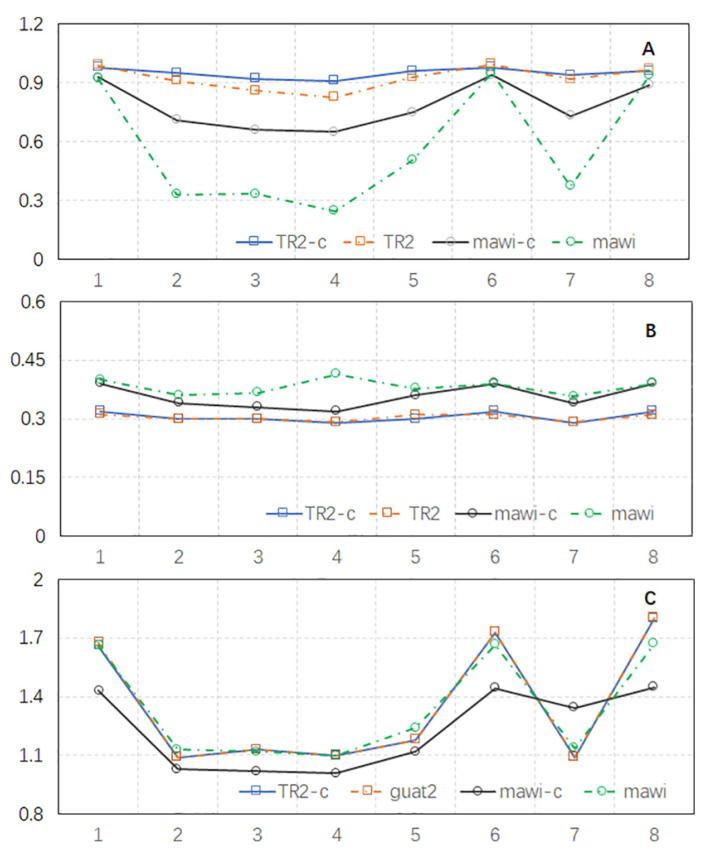
The three objectives when $\left[T_{initial},r,P_{elephant}\right]$ is as listed in Table 6 after packet completion. (**A**) The F1 score of elephant prediction. (**B**) The elephant prediction efficency. (**C**) The network latency increase.

More packets forwarded to controllers in our proposed approach means more interaction between the controllers and the switches, leading to increased network latency. Increasing the value of *r* can effectively reduce the number of flow packets forwarded to the controllers and maintain the overall network latency. However, this results in a decrease in prediction accuracy, as shown in Figure 6A. To address this issue, we propose a packet completion approach that improves elephant prediction accuracy while limiting the number of packets forwarded to the controllers. Our approach involves inserting packets between two packets received by the controllers. We set the initial timeout to 0.00001 s and used the inter-arrival time of the first two packets forwarded to the controllers ($t_{12}$) to represent the actual packet inter-arrival time at the switches. Note that $t_{12}$ may not accurately represent the inter-arrival time of packets in a flow at the switches and is instead used for packet completion. Let $p_{current}$ denote the size of the packet just received by the controllers and $t_{ctimeout}$ denote the current timeout of the flow entry. The ratio $t_{ctimeout}/t_{12}$ indicates the number of packets that should be inserted, with each packet having a size of $p_{current}$. The packet completion approach was applied to the TR2 and MAWI datasets. The prediction accuracy, efficiency, and network latency increase for each adjustment were recomputed, and they are listed in Table 6. The results are displayed in Figure 7.

It is apparent that the proposed packet completion approach can improve the prediction F1 score for both TR2 and MAWI, especially when *r* is greater than one, as shown by the F1 scores over labels 2, 3, 4, 5, and 7 in Figure 7A. When applying the same adjustment strategy over the datasets, the datasets with packet completion achieved a slightly improved prediction efficiency and latency increase. Since we could apply the approach with larger *r* values over the datasets with packet completion to achieve a similar high prediction F1 score to applying the approach with r=1 over the datasets without packet completion, the latency increase could be reduced due to a larger *r* value. More intelligent data completion approaches can be applied to further improve the prediction accuracy, efficiency, and latency increase in our future work.

#### 5.4.3. Others

Since a short initial timeout of 0.00001 s is given to flow entries until flows are out of their lifespans or identified as elephants, flow entries with short lifespans can be timed out, and the flow table that is used to store such flow entries can be free for other flows, leading to effective flow table usage. However, short timeouts can cause packets over high-performance networks such as TR1, TR2, and MAWI to arrive at controllers very frequently, especially in the early stages of flows. That may add extra overhead for the controllers to quickly complete the prediction before the next packet arrives. To reduce this overhead, controllers can perform the prediction for each flow after the first 3 or 4 packets have been received. In high-performance networks with 10G+ link bandwidth, our future work should investigate how to avoid packets with 1.5+K bytes when generating traces and how such packets affect the elephant model and prediction. Similar approaches can also be used to model and predict IoT and DDoS flows without adding dedicated devices over SDNs.

## 6. Conclusions

This paper proposed an elephant prediction strategy in SDNs. The strategy uses the timeouts of flow entries to sample packet traffic and accurately predict elephants in the early stages while maintaining the overall network latency. By applying LR to the carefully selected flow features, two sets of elephant models were trained on the TR1 dataset. One was a general model for both TCP and UDP elephants, and the other, consisting of two submodels, was dedicated to TCP and UDP elephants. Since the models were trained on the complete dataset, while the real traffic used to predict elephants was sampled by the timeouts of flow entries, we formulated an optimization problem that found the best initial timeouts of the flow entries, the rate at which the timeouts varied, and the probability threshold for elephant prediction to maximize the accuracy and efficiency of the elephant prediction while maintaining the network latency increase. The BO algorithm was applied to solve the problem.

Seven datasets were collected from different campus, backbone, and IoT datasets to estimate the robustness of the proposed models and the parameters of the timeouts. The results show that the proposed models could achieve a prediction F1 score greater than 0.9 over all the collected datasets. The proposed models and timeouts could efficiently predict elephants in about half of their life spans. However, they may lead to a widely varying increase in network latency over networks according to their mean packet inter-arrival times. Networks with long mean packet inter-arrival times, such as UNIBS, UNIV1, Simpleweb, and the IoT, can significantly reduce controller-switch interaction, leading to significantly reduced network latency. Networks with short mean packet inter-arrival times, such as TR1, TR2, and MAWI, may forward more traffic to controllers when applying our proposed approach to predicting elephants. The volume of traffic forwarded to the controllers may be larger. However, when using a larger *r* together with a finely tuned probability threshold could reduce the volume of traffic forwarded to the controllers, the prediction accuracy could be decreased. Our sensitivity experiments show that giving a shorter initial timeout to flow entries and keeping it short until the flows have been predicted to be elephants or out of their lifespan can maintain a high generalization accuracy over many networks. However, a simple packet completion method that carefully inserts packets into the packet series of flows received by the controllers can increase the prediction accuracy while limiting the increase in network latency due to packet loss. Further research on packet completion and the applications of the proposed approaches in other scenarios should be carried out in our future work.

## Figures and Tables

**Table 1:** Elephant prediction approaches comparison. FL = flow level; PL = packet level; A = accuracy; B = bandwidth cost; D = network delay.

Work	Location	Model	Traffic Sampled and How	A	B	D
[13]	controller	FL	not	high	high	mid
[14]	FPGA	FL	not	high	-	low
[15]	controller	PL	using first 5 pcks	mid	mid	low
[16]	controller	PL	using first 10 pcks	mid	mid	low
[17]	host	FL	not	high	high	mid
[18]	host	FL	not	high	high	mid
[22]	switch	FL	not	high	high	mid
[19]	switch	FL	not	high	high	mid
[20]	controller	FL	not	high	high	mid
[21]	controller	FL	sampling based on ISN	high	mid	mid
[23]	controller	FL	sampled by sFlow	high	mid	mid
[24]	controller	FL	sampled by sFlow-RT	high	mid	mid
[25]	hybrid	FL	Sampling and pick algorithm	high	mid	low
[26]	hybrid	FL	not	high	mid	low
this work	controller	FL	flow entry timeout	high	low	mid

**Table 2:** ML-based approach comparison. DRL = deep reinforcement learning.

Objective	Works	ML	XAI	Method
traffic classification	this work	LR	Yes	predict elephants
		BO	Yes	tune timeout value
	[32]	SVM	No	classify UDP flows
	[31]	DNN	No	classify elephants
	[26]	DT	No	detect elephants
	[36]	GA	Yes	feature selection
	[37]	RF	Yes	intrusion detection
	[38]	DT	Yes	intrusion detection
flow table management	[29]	RF	No	predict entry duration
	[33]	Markov	No	predict entry duration
	[10]	Q-learning	No	select entry timeouts
routing optimization	[27]	DRL	No	optimize parameters
image identification	[35]	DNN	Yes	COVID-19 diagnose

**Table 3:** Elephant models and F1 scores under a fixed hard timeout of 0.00001s.

Scenario	$W_{i}\left(10^{-4}\right)$	*b*	F1 Score (TR1)	F1-Score (Sampled)
1	[1.66101,32.2325,−1.1509]	−32.2119	0.99+	0.89
2 (TCP)	[−2.9942,28.3122,−34.2648]	−27.2731	0.99+	0.89
2 (UDP)	[−4.3848,26.0208,−30.15]	−25.6452	0.99+	0.89

**Table 4:** Symbols and their descriptions.

Symbol	Description
$T_{initial}$	original timeout of flow entries
*r*	timeout increase rate
$P_{elephant}$	probability threshold for prediction
F1	elephant prediction F1 score
Rec	elephant prediction recall
Pre	elephant prediction precision
*E*	elephant prediction efficiency
*L*	controller–switch interaction
*F*	flow set
*i*	flow *i* in *F*
*j*	the jth packet a flow forwards
$K_{i}$	number of packets flow i forwards
$label_{i}$	label of flow *i*
$plabel_{i}$	the final predicted label of flow *i*
$T_{i}$	life span of flow *i*
$b_{ij}$	the *j*th packet of flow *i* forwarded or not
$t_{ij}$	the arrival time of the *j*th packet of flow *i*
$tactivate_{i}$	the recently activate time of flow *i*’s entry
$tcall_{i}$	the recently call time of flow *i*’s entry
$Tcurrent_{i}$	the current timeout of flow *i*’s entry
$C_{\left[T_{initial},r,P_{elephant}\right]}$	total packet counts forwarded
$C_{baseline}$	total packet counts forwarded at base line
*S*	domain of $\left[T_{initial},r,P_{elephant}\right]$
$T_{shortest}$	shortest mean packet inter-arrival time
$s_{i}$	a sample in BO
$f(s_{i})$	the objective function in BO
*D*	samples and their costs in BO

**Table 5:** Traces (5 min) used for evaluation. MAWI only includes the IPV4 flows. TF, UF, TE, and UE are the short forms of TCP flows, UDP flows, TCP elephants, and UDP elephants, respectively.

Traces	TR1	TR2	Simpleweb	UNIBS	UNIV1	MAWI	IoT
Type	campus	campus	campus	campus	campus	backbone	IoT
Coun.	China	China	Netherlands	Italy	US	Japan-US	Australia
Year	2020	2021	2003	2009	2009	2013	2016
Flows	571,009	803,315	70,116	265	7562	504,637	187
TF	0.53	0.74	0.86	0.68	0.42	0.79	0.45
UF	0.47	0.26	0.14	0.32	0.58	0.21	0.55
TE	0.37	0.69	0.988	1	0.97	0.94	0.66
UE	0.63	0.31	0.012	0	0.03	0.06	0.34
Pcks/s	70,200	77,050	2070	83.99	2800	92,650	5

**Table 6:** Parameters optimized by BO algorithms under various domains. D1 is the domain of $T_{initial}$, D2 is the domain of *r*, and D3 is the domain of $P_{elephant}$.

Num.	TCP/UDP Model	D1	D2	D3	$T_{\mathrm{initial}}$	*r*	$P_{\mathrm{elephant}}$
1	same	[0.00001, 5]	[1, 5]	[0.2, 0.7]	0.00001	1.047	0.7
2	same	[0.00001, 5]	[1, 5]	[0.2, 0.8]	0.00001	2.6674	0.8
3	same	[0.00001, 5]	[1, 5]	[0.2, 0.9]	0.00001	2.798	0.2
4	same	[0.00001, 5]	[1, 5]	[0.2, 1.0]	0.00001	3.19	0.2
5	various	[0.00001, 5]	[1, 5]	[0.2, 0.7]	0.00001	1.86	0.7
6	various	[0.00001, 5]	[1, 5]	[0.2, 0.8]	0.00001	1	0.566
7	various	[0.00001, 5]	[1, 5]	[0.2, 0.9]	0.00001	2.589	0.9
8	various	[0.00001, 5]	[1, 5]	[0.2, 1.0]	0.00001	1	0.2

## Data Availability

The dataset TR1 and TR2 are not available due to privacy. The references or links for the other datasets used in this paper have been given.

## Abbreviations

The following abbreviations are used in this manuscript:

SDNs	Software-defined networks
IoT	Internet of Things
QoS	Quality of service
BO	Bayesian optimization
ML	Machine learning
TCP	Transmission Control Protocol
UDP	User Datagram Protocol
TCAM	Ternary content-addressable memories
DDoS	Distributed denial-of-service attack
BT	Bayes’ theorem
NN	Neural network
DNN	Deep neural network
DT	Decision trees
DRL	Deep reinforcement learning
RL	Reinforcement learning
SVM	Support vector machines
LR	Logistic regression
RF	Random forest
XAI	Explainable artificial intelligence
GA	Genetic algorithm
CDF	Cumulative distribution function
GPR	Gaussian process regression
